# Insights on Cryogenic Distillation Technology for Simultaneous CO_2_ and H_2_S Removal for Sour Gas Fields

**DOI:** 10.3390/molecules27041424

**Published:** 2022-02-19

**Authors:** Tengku Nur Adibah Tengku Hassan, Azmi Mohd Shariff, Mohd Mu’izzuddin Mohd Pauzi, Mai Syadiah Khidzir, Amiza Surmi

**Affiliations:** 1 CO_2_ Research Centre (CO_2_RES), Institute of Contaminant Management, Universiti Teknologi PETRONAS, Bandar Seri Iskandar 32610, Malaysia; tengku_19000950@utp.edu.my (T.N.A.T.H.); mohd.muizz@utp.edu.my (M.M.M.P.); msyadiah.khidzir@utp.edu.my (M.S.K.); 2 Chemical Engineering Department, Universiti Teknologi PETRONAS, Bandar Seri Iskandar 32610, Malaysia; amiza_surmi@petronas.com.my; 3 Group Research & Technology, Petroliam Nasional Berhad (PETRONAS), Lot 3288 & 3289, off Jalan Ayer Itam, Kawasan Institusi Bangi, Kajang 43000, Malaysia

**Keywords:** natural gas, high CO_2_ separation, H_2_S removal, conventional cryogenic technology, unconventional cryogenic technology

## Abstract

Natural gas demand has dramatically increased due to the emerging growth of the world economy and industry. Presently, CO_2_ and H_2_S content in gas fields accounts for up to 90% and 15%, respectively. Apart from fulfilling the market demand, CO_2_ and H_2_S removal from natural gas is critical due to their corrosive natures, the low heating value of natural gas and the greenhouse gas effect. To date, several gas fields have remained unexplored due to limited technologies to monetize the highly sour natural gas. A variety of conventional technologies have been implemented to purify natural gas such as absorption, adsorption and membrane and cryogenic separation. The application of these technologies in natural gas upgrading are also presented. Among these commercial technologies, cryogenic technology has advanced rapidly in gas separation and proven ideally suitable for bulk CO_2_ removal due to its independence from absorbents or adsorbents, which require a larger footprint, weight and energy. Present work comprehensively reviews the mechanisms and potential of the advanced nonconventional cryogenic separation technologies for processing of natural gas streams with high CO_2_ and H_2_S content. Moreover, the prospects of emerging cryogenic technologies for future commercialization exploitation are highlighted.

## 1. Introduction

In 2020, BP reported the sharp decline in global natural gas consumption by 81 billion cubic meters (bcm), or 2.3%, due to the unprecedented impact of the COVID-19 outbreak and oil crisis leading to economic downturn worldwide [1]. Despite the persistent impact of the crisis, the U.S. Energy Information Administration (EIA) projected a growth of natural gas utilization by 30% between 2020 and 2050 with the uplifting of economic activity and travel restrictions [2]. In fact, the outlook for natural gas demand seems to be more resilient compared to oil demand. The natural gas demand is expected to recover and robustly increase to approximately 5300 bcm by 2050 [3]. The rising demand of natural gas is mainly driven by progressive economic activities and industrialization in developing Asian countries, such as China and India, with the transition from coal to low-carbon fuels. Hence, the utilization of natural gas is anticipated to continue outperforming the consumption of fossil fuels for power generation and transportation fuel in the future, as it has a lower price and carbon footprint [1,4,5,6,7]. Natural gas, however, is a fossil fuel that is extracted from deep beneath the earth’s surface and primarily consists of hydrocarbons or higher alkanes such as ethane, propane, butanes and pentanes. It also contains trace amounts of nonhydrocarbons or impurities, including sulfur, helium, nitrogen, hydrogen sulphide and carbon dioxide, and it must be retained as sweet gas for market sale. Natural gas with hydrogen sulphide, having a H_2_S content of more than 4 parts per million (ppm), is considered sour gas. Sour gas obtains its name from the fact that it smells like rotten eggs and can be found naturally in sewers, crude, hot springs and volcanoes rather than being created by industrial processes such crude oil and gas refining, wastewater treatment and pulp and paper manufacturing. As a result, some underdeveloped gas resources have remained unknown. China has developed the majority of sour gas reservoirs, with about 60% of gas fields containing 20,000 to 40,000 ppm H_2_S and some containing more than 100,000 ppm [8]. It was stated that the higher the level of H_2_S, the deeper the reservoir, subsequently leading to a higher temperature, which encourages thermochemical reaction [9]. With the deposition of sulfur, the formation of these gases in natural gas can clog and damage pipelines. During the industrial process, any leakage or blowout of this gas will severely harm the well site and pollute the environment. Furthermore, most of the research solely focuses on CO_2_ removal to overcome the challenges mentioned before. However, there are only a few studies available that study simultaneous CO_2_ and H_2_S removal.

As a result, to solve the pipeline problem, this sour gas must go through a sweetening process to remove H_2_S and CO_2_. Absorption, adsorption, membrane separation and cryogenic distillation are the existing technologies for natural gas sweetening. The process that has been widely used for natural gas sweetening is absorption. The absorption process can be classified into two types, which are chemical absorption and physical absorption. These processes are explained in detail in Section 2.1. Amine gas treatment is one of the examples of chemical absorption technology that is widely used in the pretreatment unit of LNG plants and other gas processing plants to sweeten the gas. This process uses amines or other solvents to absorb the acid gases to remove the sour gases from the natural gas. However, this process is not economically viable for high concentrations of gas, as it requires a large footprint and complex offshore facilities. It also requires solvent transportation in order to operate at offshore facilities.

Nevertheless, there are still other technologies that have been developed and implemented in the industry which are more economical and practical. Instead of implementing the absorption process, the adsorption process is applied at industrial scale for a more cost-effective removal [10]. However, Cherif [11] stated that the dominating market share of the adsorption process started to decrease in 2008 due to the development of other technologies. This is because this technology requires a treatment process for the final product. In addition, the operation and expense increase significantly as the supply of fossil fuels decrease [1]. The adsorption technology is reviewed in more detail in Section 2.2. Apart from adsorption, membrane separation can be used to separate the majority of CO_2_ quickly, but it has many drawbacks in terms of operating costs, heat and chemical resistance and long-term process stability [12]. To obtain less than 2% CO_2_ in gas sales, this technology necessarily requires gas staging and recycling [13]. This technology is explained in more detail in Section 2.3.

Out of these technologies, cryogenic distillation technology is in a mature stage of its life cycle and has been routinely used in medium to large-scale plants to produce nitrogen, oxygen and argon. This technology is preferred, as it is the most cost-effective technology for high CO_2_ concentrations and high production rate plants to purify a very high content of oxygen and nitrogen. By reducing the temperature to lower than 73.3 °C using a refrigeration system, cryogenic distillation converts H_2_S and CO_2_ from the gas phase to the liquid or solid phase and then valorizes the product [11]. The main advantage of this process is it can produce liquid CO_2_ that is ready for transportation. One feature of this technique is that it does not involve any water or solvent, which would raise the expense of the removal process. 

Overall, natural gas consumption will continue to rise significantly in the coming decades, outpacing that of other fossil fuels. The growth demands are likely to turn into the exploitation of more sour gas fields and the development of new technologies to separate H_2_S and CO_2_ gas from natural gas. Therefore, this paper critically reviews the conventional separation technologies that are applied in industries, focusing more on the insights into cryogenic separation. Cryogenic distillation technologies give more competitive benefits compared to other technologies such as amine gas treatment and membrane separation. Cryogenic distillation has low energy consumption and low CO_2_ content in the feed stream when compared to amine treatment [11]. The insights of this technology for future removal of sour gas from natural gas are reviewed in more detail in Section 3. The important insights of the review on the conventional separation technologies and advanced cryogenic process for CO_2_ and H_2_S removal from sour natural gas are summarized in Table 1.

## 2. CO_2_ and H_2_S Separation Technologies

### 2.1. Absorption

At the beginning of the 21st century, the first solvent that was used for the absorption process was a carbonate solution that was applied in dry ice plants to separate CO_2_ from flue gas [14]. Sodium carbonate solutions were rapidly phased out after the introduction of alkanolamine, as this solvent absorbs CO_2_ faster and can attain extremely high CO_2_ removal efficiency [14]. Starting in 1930, primary generation alkanolamine solvents such as diethanolamine (DEA), (MEA) and diglycolamine (DGA) were developed for the CO_2_ removal process [14,15,16]. Then, secondary alkanolamine solvents such as diethanolamine (DEA) and diisopropanolamine (DIPA) were invented as alternatives to MEA [17]. The difference between primary and secondary is that the primary alkanolamines contain hydrogen atoms directly bonded to nitrogen [17]. A tertiary alkanoamine solvent, which is methyldiethanolamine (MDEA), was proposed by Frazier and Kohl to promote the selectivity of H_2_S [17]. In 1983, 2-amino-2-methyl-1-propanol (AMP), which hindered amines, was developed at Exxon Research and Engineering Company [18]. From 1995 to now, there has been a lot of research into sterically hindered amines as potential alkanol amine absorbents. There are also lots of studies that report the promoters, such as piperazine, potassium carbonate, mono ethanol amine (MEA) and diethanolamine (DEA), that can be blended with amine solvent, such as MDEA, to enhance the reaction rates of the absorption process [17]. Starting in 2005, the interest in the usage of amino acid for acid gas removal started to develop in using the sodium or potassium salt glycine (NaGly), which is the simplest primary amino acid, for CO_2_ capture [19]. Recently, in 2003, ionic liquids, ILs, were branded as “solvents of the future”, as they have the potential as alternative solvents for acid gas removal [20,21].

**Figure 1:**
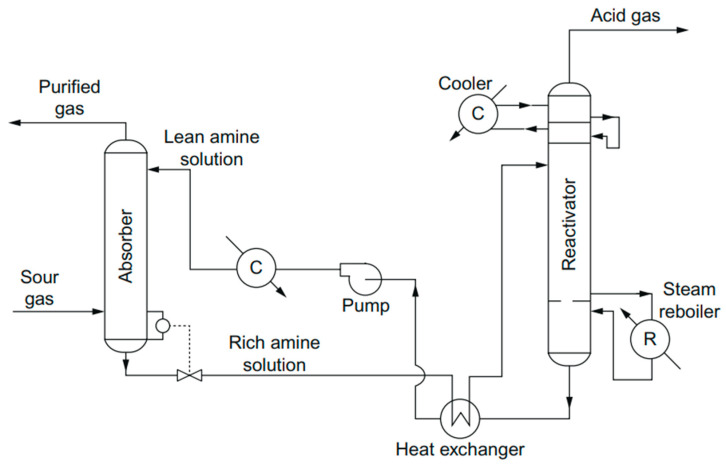
Schematic diagram of the amine sweetening process [22]. Reprinted with permission from Speight, J.G. (2015). Copyright 2021 Elsevier.

The absorption process is the process where a gas mixture comes into contact with a liquid (solvent). The gas phase is transported into the liquid phase in the absorption process. This process has been used in the natural gas industry for 100 years for the gas sweetening process to separate sour gas from natural gas. It is used to remove any impurities or contaminants in gas mixtures to recover valuable products. This process can be classified into two types of solvents, which are chemical solvents and physical solvents. In chemical solvent processes, alkanolamines or alkaline salts with weak acids, such as sodium or carbonates, are used as solvents for the absorption process. The sweetening of sour gas via absorption is referred to in Figure 1 by using alkanolamines. The sour gas is injected through a tower and makes contact with alkanolamine solutions for the absorption process to happen. The solvent absorbs sulfur compounds from H_2_S and release the effluent gas. Then, the desorption process, or regeneration process, is performed to strip the acid gases from the solvent at low pressure or high temperature. 

**Figure 2:**
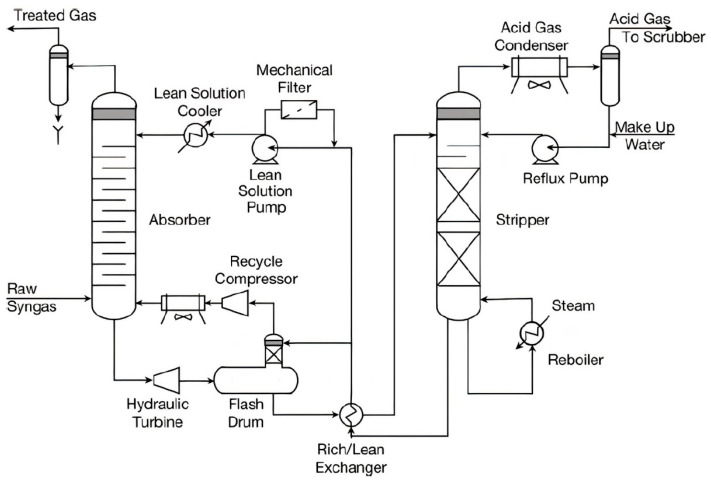
Schematic diagram of common physical absorption process (Selexol process) [24,25]. Reprinted with permission from Miller, B.G. (2011). Copyright 2021 Elsevier.

In physical solvent processes, organic solvents such as methanol, N-Methyl-2-Pyrrolidone, Poly(Ethylene Glycol) Dimethyl Ether, Sulfolane and Diisopropanolamine are used to absorb H_2_S and CO_2_ depending on the partial pressure. The performance of the absorption is increased at high partial pressure and low temperature. This process does not have any corrosive effects and also does not require the addition of heat in the stripping process. In addition, physical solvents can strip off impurities without any additional heat. However, this process is not suitable for bulk absorption of CO_2_ gas, as it requires high pressure and low temperature [23]. Figure 2 shows the flow diagram for the physical absorption process.

In chemical absorption, an aqueous solution of alkanolamines is one of the most effective absorbents that is still widely used for the absorption process for natural gas purification. Nevertheless, this solvent has many drawbacks, including foaming, high cost, high energy consumption for regeneration, high toxicity and high corrosivity. The drawbacks faced by amine-based solvents can be catered back by using aqueous amino acids, as they have the same functional group. Amino acids are one of the alternative solvents that have many advantages, such as high stability towards oxidative degradation, high chemical reactivity with carbon dioxide and low vapor pressure [26]. Due to their physical properties, amino acid solvents have high reactivity towards CO_2_ compared to conventional solvents such as MEA and MDEA [27]. Hu G et al. stated that amino acids such as lysine, proline and sarcosine have a larger reaction than monoethanolamine (MEA) for the absorption of CO_2_ [26]. The absorption of CO_2_ is influenced by the ionic strength, pH and cations of weak bases such as sodium and potassium for the amino acid to react with the CO_2_ gas [26]. It was also reported that the use of amino acids blended with amine shows better absorption of CO_2_ than the neutralization of amino acids with potassium hydroxide [28]. Nevertheless, the combination of these solvents for large-scale absorption application is not economical because it requires higher regeneration temperature, as stated in Erga et al. [29]. Knuutila et al. [14] found that the use of sarcosine requires a higher energy cost compared to MEA.

Ionic liquids (ILs), particularly functional ILs, are known as unique absorbents for the removal of H_2_S from gas mixtures because they can absorb a large amount of H_2_S at high pressure. Ionic liquids (ILs) have been widely studied for CO_2_ and H_2_S absorption, as they have several advantages, such as environmentally friendliness, extremely low vapor pressure, tuneable structure and high thermal and chemical stability [30]. Most of the research found that functionalized ILs can act as catalysts or substitutes for amino-based, metal-based, substituted benzoate-based and pyridinium-based solvents for H_2_S removal. However, the major drawbacks of IL-based solvents are high cost, high viscosity, complicated synthesis process and that they cannot be applied to a large-scale industry. In addition, most industries still prefer gas–liquid systems because this process requires low cost and low energy consumption during solvent regeneration. Overall, the previous literature reviews show that CO_2_ and H_2_S removal using green solvents such as amino acid and ionic liquid are not economical for commercial application due to high cost and energy consumption during operation and regeneration. Moreover, most of the studies for green solvents such as amino acid only focus on CO_2_ removal, whereas there are only a few literature studies about H_2_S removal [30].

### 2.2. Adsorption

The first systematic research on adsorption application began in 1773, conducted by Schelee to observe the adsorption of air by using charcoal via a volumetric apparatus [31]. The first commercial application of the adsorption process was purifying white sugar. Back in 1783, charcoal had been used as the adsorbent to remove impurities and contaminants from sugar [32]. However, during that era, people were not concentrating on the improvement of adsorbent properties and only relied on the natural adsorbent properties such as charcoal, clay and peanut hull [33]. In the 19th century, the study to improve the properties of adsorbent started along with the industrial revolution. The researchers concentrated on the surface area and the porosity of the adsorbent [34,35]. The idea to improve the adsorbent based on the porosity and the surface area began after Chappius measured the isotherm from the adsorbent layers [36].

Adsorption has had numerous applications in industry in the past 30 years, such as in the purification of gas mixtures, mainly in petrochemical, environmental, electronic and medical industries, due to its unique characteristics [37]. There are many types of adsorbents available with different pore sizes and selectivity that create flexible designs to separate and purify gas mixtures for particular desired goals. The impurities removal application includes organic and inorganic impurities removal, electronic gas purification, air pollution control, gas drying, solvent vapour recovery and nuclear waste management. Moreover, the gas separation application includes methane and carbon dioxide separation, hydrogen recovery, air separation, alcohol dehydration, production of ammonia synthesis gas and isoparaffin separation [38]. The well-known technologies commercially used are pressure swing adsorption (PSA), temperature swing adsorption (TSA), vacuum swing adsorption (VSA) and electric swing adsorption (ESA) [39,40]. For NG application, TSA is used for gas purification, whereas bulk gas separation often uses the PSA process [37].

PSA technology was first patented in 1932 by Charles Skarkstrom for oxygen enrichment, in which the cyclic adsorption process was employed using four main steps that included feed, blowdown, purge and pressurization [41]. In PSA, the acid gas stream that contains high CO_2_ makes contact with spherical adsorbents packed in the column which are typically arranged in parallel to maximize the energy efficiency [42]. The feed gas is initially fed into the column and pressurized to a pressure higher than atmospheric pressure. CO_2_ is selectively bound and adsorbed on the surface of the adsorbent at high pressure and low temperature until equilibrium is achieved. The adsorbent is saturated with adsorbed CO_2_. Hence, the regeneration of the adsorbent takes place by restricting the gas flow and depressurizing the column to liberate the CO_2_ from the surface of the adsorbent [43]. In contrast, the regeneration of adsorbent in VSA is performed at vacuum condition by reducing the pressure below atmospheric pressure, whereas in TSA, CO_2_ is liberated by increasing the temperature at a constant pressure [44,45,46]. Meanwhile, ESA is named due to the low-voltage electric current being introduced to heat the adsorbent by the direct Joule effect [47]. The difference between TSA and ESA is that ESA heats the adsorbent by using electric power while TSA heats the adsorbent by using waste heat from noncondensable gas [48].

There are a wide range of adsorbents that have been utilized according to their level of selectivity attraction towards contaminants to be removed. Furthermore, adsorbent enhancement could be achieved by synthesizing and impregnating other compounds onto the surface of the adsorbent. There are two types of adsorption approaches, either physisorption or chemisorption. The physisorption process is a physical process directed by weak Van Der Waals bonds between the adsorbent and adsorbate [49]. On the other hand, chemisorption is governed by the bond formation between the functional groups that attach at the surface of the adsorbent and the adsorbate [49]. Due to the attached functional groups, chemisorption is generally more efficient than physisorption, specifically when using basic and oxygen-containing groups, as CO_2_ is acidic in nature; hence, these functional groups play a major role in the removal of CO_2_ from natural gas [50]. The specific surface area of the adsorbent also has a major effect on the adsorption process. It is well-known that the higher the specific surface area, the higher the adsorption capacity. The major disadvantage of the adsorption system is waste generation. One of the solutions to overcome this issue is introducing the regeneration process to the adsorbent. However, regeneration may not be effective in the long run. The highest number of regeneration cycles obtained in the literature is 5 cycles, after which removal efficiency starts to decrease [51].

The interest in H_2_S removal in natural gas processing units enhanced several studies to develop regenerable solid sorbents. However, the low adsorption capacity of commercial solid adsorbents needs a large amount of adsorbent bed while frequent disposal of saturated adsorbents is one of the major environmental concerns. The adsorbent-filled fixed bed reactors for H_2_S capture may operate according to two different mechanisms, either physisorption or chemisorption [52]. Zinc and copper oxides supported on porous adsorbent silica have attracted recent attention for H_2_S capture due to the potential for the combination of sulfidation thermodynamics and active metal oxides [53,54]. The porous adsorbent is generally synthesized from various composite materials based on metal foams [55], zeolites [56], laterites [57], kaolin [58], silica [59], carbon of miscellaneous sources [60] and other solid phases impregnated with metal salts and their mixtures in order to enhance the adsorption capability of the fixed bed. Bahman Elyasis et al. stated that Cu-ZnO nanoparticles impregnated with mesoporous silica show a significant impact on H_2_S removal. The amount of H_2_S captured is 75 mg/g_sorbent_, while the commercial ZnO only captures about 34 mg/g_sorbent_ [61]_._

NG often contains significant amounts of CO_2_ and H_2_S that have to be reduced to less than 1% for CO_2_ and 4 ppm for H_2_S to meet the fuel gas specifications for pipeline transportation [62]. Adsorption also has been recognized to be an energy-efficient technology for CO_2_ and H_2_S removal, provided that the material exhibits high and stable adsorption capacity along with excellent selectivity toward acid gases. The early work for simultaneous adsorption separation of CO_2_ and H_2_S from methane was performed by Huang et al. by using amine impregnated on silica xerogel. They found that the excellent performance in terms of CO_2_ and H_2_S adsorption by amine-modified silica is due to large amounts of amine groups on the surface resulting from their high surface areas [63]. However, the CO_2_ and H_2_S selectivity was not mentioned. Recently, Ma et al. reported that polyethylenimine (PEI) impregnated with silica is capable of selective adsorption of both CO_2_ and H_2_S. However, it was stated that not only is the adsorption of CO_2_ diffusion limited, but also the optimum temperature for CO_2_ adsorption is 75 °C, whereas the H_2_S is 25 °C. Moreover, CO_2_ seems to strongly inhibit the H_2_S adsorption at room temperature [64].

The usage of amines as activators for the adsorbent demonstrates certain limitations to CO_2_ and H_2_S separation because by nature the CO_2_ has higher acidity than H_2_S. Due to both CO_2_ and H_2_S being electron acceptors, they have a strong effect on the amines group which leads to reactive and nonreactive adsorption, hence restricting the efficiency of CO_2_ and H_2_S separation. This phenomenon occurs due to the adsorbent being more selective toward H_2_S than CO_2_ [10]. Most of the commercial gases used in industries consist of both acid gases, such as CO_2_ and H_2_S, and polar species, such as H_2_O or CO. A study performed by Billow et al. investigated the reaction of CO_2_ and H_2_S adsorption on LTA and FAU zeolite. They found that H_2_O was the highest preferred by the adsorbent, followed by H_2_S and CO_2_ [65]. Hence, a proper study needs to be conducted to investigate the selectivity ratio of H_2_O, CO_2_ and H_2_S for better separation efficiency.

### 2.3. Membrane

Over the past few decades, membrane technology emerged as a sustainable process in various applications such as wastewater treatment, food technology, medicine, pharmaceuticals and petrochemicals [66]. In the 1980s, the first commercial membrane technology was introduced by Air Product for air and hydrogen production using polysulfone-based hollow fiber membrane [67]. Presently, membrane technology continuously evolves as a mature and competitive technology that contributes to 10% of the market share in natural gas purification [68]. According to Mordor Intelligence [69], the gas separation membranes market will progress at a compound annual growth rate (CAGR) of 5% between 2020 and 2025. The expansion of the membrane separation market is fueled by the growing demand for CO_2_ removal attributed to the rising environmental concerns and low monetization of sour gas fields. Membrane-based acid gas removal technology is ideal for the purification of highly sour natural gas with CO_2_ concentration of more than 50 mol% and H_2_S up to 10 mol% [70]. Moreover, the simplicity of the membrane process enhances its potential for installation at offshore platforms for gas processing with a low flow rate of <6000 Nm^3^/h [71]. The emerging development of membrane technology is rendered by its flexibility for scale-up, small footprint and high energy efficiency. Compared to absorption, membrane separation is more economical and environmentally friendly, as it does not require the utilization of any chemical solvent, which eliminates the need for compression to regenerate solvent.

**Figure 3:**
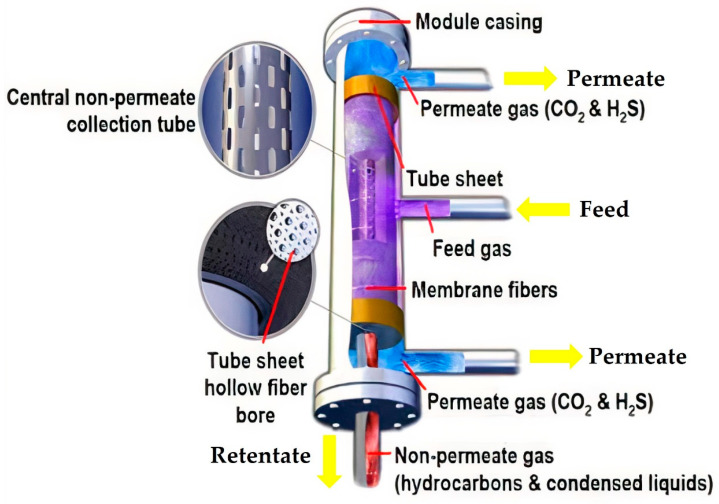
Illustration of selective separation of CO_2_ and H_2_S from natural gas using hollow-fibre membrane module [74]. Reprinted with permission from Sanghani, P. et al. (2020). Copyright 2021 Society of Petroleum Engineers (SPE).

Membranes are generally classified based on material (e.g., glassy or rubbery polymers) and configuration (e.g., flat sheet, hollow fiber or spiral wound). Gas separation is achieved through selective gas transport across the semi-permeable barrier. Figure 3 shows the selective separation of CO_2_ and H_2_S from natural gas using a hollow fibre membrane system. Gas transport in membrane separation is driven by the pressure and concentration gradient. Theoretically, gas transport behavior in membrane separation is governed by the solution–diffusion mechanism where solubility (S) and diffusivity (D) play important roles to determine gas permeability. The types of membrane material and properties of the permeants, such as condensability, influence gas transport across the membrane. In addition, the molecular size of the gas molecules significantly affects the gas diffusion rate in the order of C_3_H_8_ > C_2_H_6_ > CH_4_ > N_2_> O_2_ > CO_2_ > H_2_S > H_2_ [72]. Membrane separation also requires sufficiently high pressure to drive gas separation. In addition, the pretreatment process is required prior to gas separation to remove any water or heavy hydrocarbons from the feed gas to avoid membrane fouling [73].

Commercial membranes such as cellulose acetate [75], polyimide [76,77] and polyamide are the leading materials used in membrane gas separation due to their robustness, superior permeability and selectivity, as well as excellent durability. Glassy membranes such as cellulose acetate are typically effective for CO_2_ separation, while rubbery membranes are typically effective for H_2_S separation. Cyanara-NATCO [75] developed a cellulose triacetate (CTA)-based hollow fiber membrane system for natural gas sweetening in the offshore processing facility at the Cakerawala platform located in the Gulf of Thailand. The membrane system is capable of handling 228.89 m^3^/s process gas and successfully reduces the CO_2_ content from 37% to 15%. Recently, Nafisi et al. discovered the potential of fluorinated polyimides (6FDA) for CO_2_ separation, where CO_2_ permeability attained as high as 1468 Barrer with CO_2_/CH_4_ selectivity of 22.6 [78]. Besides polymeric membranes, zeolite membranes such as silicalite-1 [79], SAPO-34 [80,81] and DDR [82,83] also demonstrated a promising performance for separation of CO_2_ from CH_4_. Zeolite membranes are known for their high selectivity, which is attributed to the uniform pore structures. Poshuta et al. reported a separation factor of 36 with CO_2_ permeance of 2 × 10^8^/mol m^−2^ s^−1^ Pa^−1^ by using SAPO-34 membrane [84]. Furthermore, Cui et al. demonstrated higher performance of zeolite T membrane, where CO_2_ permeance and CO_2_/CH_4_ selectivity was found to be 4.6 × 10^8^/mol m^−2^ s^−1^ Pa^−1^ and 400, respectively [85]. The application of zeolite membrane is presently confined to lab-scale study due to its brittle nature and complex fabrication procedure which hamper its commercial opportunity.

On the other hand, some membrane materials also demonstrate promising capability to handle contaminants other than CO_2_, such as H_2_S. Currently, the studies on H_2_S removal using membrane separation are usually confined to low H_2_S concentration due to high toxicity and stringent safety requirement during handling. Moreover, the simultaneous existence of these impurities also leads to competitive sorption that consequently minimizes membrane separation performance [86]. Recently, polymers with an etheric oxide (EO) unit, such as commercial PEBAX^®^, appear as promising membranes that offer high H_2_S/CH_4_ selectivity ascribed to their high specific interaction with polar molecules such as H_2_S [87]. Previous studies demonstrated that PEBAX^®^ exhibit H_2_S/CH_4_ selectivity up to 80 [88,89]. Moreover, some membrane materials also have inherent copermeation properties of CO_2_ and H_2_S with relatively high selectivity over CH_4_. Cellulose acetate (CA) membrane was the first commercialized polymeric membrane since the 1980s and is presently used in the industry for acid gas removal [86]. Achoundoung et al. [90] reported the equivalent CO_2_/CH_4_ and H_2_S/CH_4_ selectivity of CA membranes up to 30 under feed pressure of 3447 kPa. However, CA membranes suffer loss of selectivity under aggressive feed conditions.

In 2017, Schlumberger in collaboration with PETRONAS successfully installed and commissioned the CYANARA PN-1 acid gas removal membrane system at onshore gas processing facilities in Terengganu Gas Terminal (TGAST), Malaysia [91,92]. The dual-zoned cellulose acetate-based hollow fibre membrane system efficiently processed 228.89 m^3^/s and produced a gas stream containing 12 mol% to 25 mol% of CO_2_ and H_2_S to meet product specifications of <8 mol% CO_2_. Compared to the conventional absorption process, the CYNARA PN-1 membrane offers economic advantages through 60% reduction in footprint and 50% installation cost savings. In addition, the installation of the membrane system also is anticipated to reduce operational expenditure (OPEX) by USD 180 million for 20 years of estimated operating cost [91]. Evonik recently launched a high-performance polyimide-based hollow fibre membrane (SEPURAN NG^®^) in 2018 for selective separation of CO_2_ and H_2_S from natural gas. However, the application of SEPURAN NG^®^ is yet to be reported elsewhere.

The application of membrane separation is usually limited to moderate gas flow rate, as performance may deteriorate at high volumes of gas processing. Multistage membrane separation may be necessary to achieve high product purity, which creates additional expenditure. Plasticization and compaction of membranes over a long operation period are the major drawbacks which may impede the excellent progress of membrane gas separation technology. The presence of impurities such as CO_2_, H_2_S and other heavy hydrocarbons may degrade the integrity of the membrane and thereby result in poor separation performance. Research methods are underway to cater these limitations through the crosslinking of polymer chains, modification of polymer properties and thermal treatment. Mixed matrix membrane (MMM) paved a new avenue in membrane development to improve their separation performance by synergistically combining the processability of polymeric membrane and high selectivity of inorganic filler.

On the other hand, the growing interest in the development of hybrid processes, which combine membrane and other conventional separation technologies, creates opportunity in gas separation. Bhide et al. [93] developed a process design study and economic assessment of hybrid processes by combining membrane separation and amine absorption, which aimed to purify natural gas that contains up to 40 mol% CO_2_ and 1 mol% H_2_S. The simulation showed that the hybrid process yielded lower operating costs, costing 1.516 MM USD/year, compared to the independent amine process, costing 2.853 MM USD/year. Furthermore, the total capital investment (TCI) of the hybrid process was estimated to be USD 4.196 MM, which was significantly cheaper than the amine process, which had a TCI of USD 6.226 MM. The cost benefits are due to the fact that the membrane removed approximately 78% of CO_2_ from the feed stream and thereby minimized the solvent circulation rate as well as the design capacity required for absorption and the solvent regeneration process. Rezakazemi et al. [94] suggested that the hybrid process is feasible for industrial gas separation with feed flow rate more than 8.17 m^3^/s and CO_2_ content higher than 12%. Moving forward, the progress of membrane technology in natural gas sweetening will focus on the development of high-performance membranes with an active layer in the order of 0.1 µm to enhance their competitiveness as a sustainable separation process.

### 2.4. Cryogenic Distillation

Numerous distillation applications were applied in the 19th and 20th centuries, particularly for the alcohol separation process [95]. In 1945, F. Taylor invented the first concept of distillation using laboratory apparatus consisting of three main pieces of equipment: a vessel, a condensing apparatus and a receiver [96]. Then, John M. Chambers (1953) established a more structured distillation column concept to purify the fermented alcohol and remove impurities from the feed stream to obtain high-purity ethanol considering the optimum reflux ratio and heat input in a more stable operation [97]. The demand for distillation technology is increasing rapidly, and the concept is growing and evolving very fast with increased opening of oil refineries and petrochemical plants around the world. Nowadays, most refineries or petrochemical plants have distillation columns in the process, and the application is not just at atmospheric pressure. Still, distillation has been expanded and upgraded for more challenging environmental conditions such as high pressure, low-temperature and high-temperature process, cryogenic temperature, vacuum condition, etc.

The distillation column is designed to separate two or more components from the feed gas stream. The concept of separation is based on differences in boiling point or relative volatility of the component. The low boiling point component is left as a top product, while the higher boiling point component is at the bottom of the column as a liquid product. The operating temperature and pressure are critical parameters in achieving the separation performance [98,99]. Typically, the distillation column operates at a two-phase region where the vapor and liquid contact plays an important role in mass transfer. The liquid will flow from top to bottom of the column, while the vapor will flow counter-currently to the top, and the vapor–liquid interaction is where the mass transfer for the separation occurs [100].

The internal design of the distillation column, including the selection of trays or packing, is key for mass transfers to meet the performance. The selection of the internals will depend on the purpose of the separation, physical properties, service or system, operating conditions, etc. Typically, the designer will conduct the process simulation to evaluate the feasibility of the design before proceeding with the detailed engineering design of the column [99,100,101,102,103,104,105,106]. The design and operation of the distillation column are challenging and require good process control to have stable operation, since it involves changes in phase inside the distillation column due to different temperature profiles in each theoretical stage. Furthermore, maintaining the operating pressure and controlling the cooling and heating rate at the condenser and reboiler are important to achieve the separation target [105,107]. Cryogenic separation is one of the most efficient methods to separate high CO_2_ concentration together with H_2_S. Cryogenic separation is widely used commercially to separate CO_2_ content to meet the pipeline specification [108].

Despite the bright potential of the technology, the operation of the distillation column is highly energy-intensive, and it consumes almost 40% of energy in the chemical industry to meet the separation performance of the process [109]. Therefore, a lot of optimization and improvement of the distillation column is currently ongoing by researchers to minimize the project’s overall cost. More advanced cryogenic technology for the process separation is further discussed in the following section.

## 3. Advancement of Cryogenic Distillation

The modifications on the conventional cryogenic distillation are further discussed in the following section.

### 3.1. Cryogenic Packed Bed

**Figure 4:**
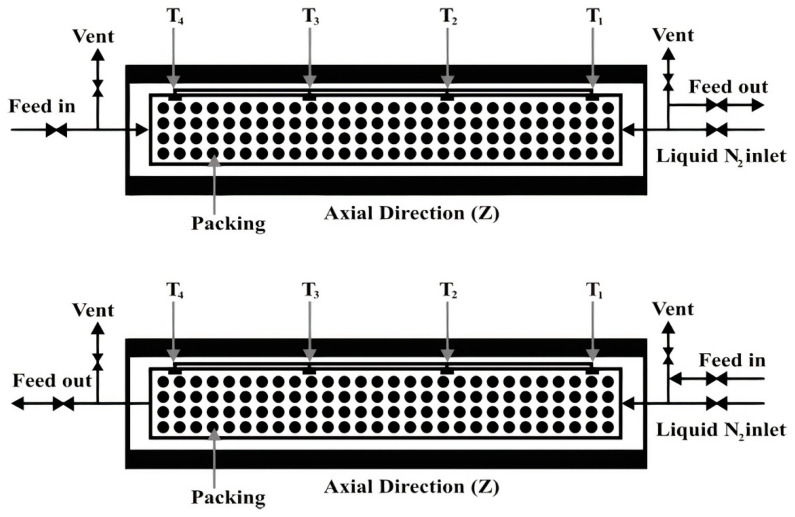
Process flow for cryogenic packed column with (above) counter current and (below) cocurrent flow configuration [111]. Reprinted with permission from Ali, A. et al. (2014). Copyright 2021 John Wiley and Sons.

A packed bed separator is one of the examples of nonconventional methods because the component will desublimate and turn into solid phase throughout the process [110]. In the CO_2_ and H_2_S capture, cold bed material is used as the heat transfer surface between the CO_2_ and the cryogenic conditions. The cryogenic separation process starts with precooling the bed material to −120 °C as a cooling step by using cold nitrogen gas that feeds into the packed bed column. After this precooling process is completed, the cold nitrogen gas is stopped from being injected into the column and switched with the flue gas containing sour gas. The CO_2_ and H_2_S sublimate and water condensates on the bed to form crystals after sufficient cooling of the bed material. These CO_2_ and H_2_S crystals continue to form on the bed material until the bed material becomes concentrated. The process of crystal formation continues on the fresh bed material further into the column. This process leads to front frost that spreads through the packed column. The rate of the front frost is called front frost velocity, and the regeneration of the packed column is required once the packed column is concentrated with the crystal [108]. In the regeneration process, CO_2_ is recycled for CO_2_ recovery while air is recycled for water recovery. The regeneration process is compatible with the low flow rate gas and needs to be performed on at least three beds in parallel. Figure 4 shows the schematic process flow for the cryogenic packed bed for CO_2_ removal [111].

The advantage of the packed bed cryogenic separation is that CO_2_, water and other impurities such as H_2_S can be simultaneously removed from the sour gas based on the differences of their dew and sublimation points [110]. In addition, the issues of pressure drop and clogging can be prevented [112]. Tuineir et al. stated that the packed bed cryogenic process has an advantage in that it does not require chemical sorbent and elevated pressure to be performed [113]. The chemical sorbent contributes to the increase in capital expenditure (CAPEX) when the chemical sorbent needs to be replaced due to the degradation effect [16]. The elevated pressure is not required on the cryogenic process due to the crystallization of CO_2_, water and H_2_S, which is determined by the cold energy in the bed material packing [113]. In addition, the packed bed cryogenic separation has better purity of the main product compared to the pressure swing adsorption process (PSA). Tuinier et al. performed a study on the effect of packed bed cryogenic separation on the quality of the methane compared to the PSA. They found that methane recovery improved to 94.3% and methane productivity improved to 350.2 kg CH_4_ h^−1^ m_packing_^3^, compared to the PSA process, where methane recovery and productivity were only about 79.7% and 43.1 kg CH_4_ h^−1^ m_packing_^3^, respectively [114].

The energy requirements for the conventional and nonconventional cryogenic methods have been questioned recently. Energy minimization is very important for technology to be economically reliable. Abulhassan et al. studied the comparison of energy requirements between the conventional method and the packed bed method, which is the nonconventional cryogenic method using gas mixture with CO_2_ concentration of 70% [111]. They found that the cryogenic packed bed used less energy, about 810 kJ/kg CO_2_, compared to the conventional cryogenic method, which used about 1472 kJ/kg CO_2_, to separate the CO_2_ from the gas mixture. In addition, a study from Tuinier et al. stated that the energy consumption for the packed bed cryogenic process was 22% lower than the PSA process [114]. The energy required for the packed bed cryogenic separation was 2.9 MJ/kg CH_4_, whereas the PSA separation process required 3.7 MJ/kg CH_4_ [114]. The CAPEX of the cryogenic process can be reduced if the methane needs to be liquefied since the methane leaving the cryogenic packed bed is already at a very low temperature; hence, the cost of the additional part to install the liquefied process system can be prevented [110].

The cryogenic packed bed faces several limitations and challenges, even though this technology has potential compared to chemical absorption and PSA. The main issue of the cryogenic packed bed is that the current thermal insulator available commercially is not good enough to maintain a low temperature during the process. The cold energy loss to the environment leads to cold energy supply increasing, along with time. Hence, the technology of the thermal insulator needs to be improved to avoid sensible and latent heat loss [115]. In addition, the simultaneous purification of CO_2_ and H_2_S requires high energy consumption, as the dew point of H_2_S is around −150 °C. For obtaining a high H_2_S removal efficiency, the process needs to be performed at a very low temperature for a long period. The process will lead to an increase in operation expenditure (OPEX) [116]. The cold energy exchange or heat integration from the liquefied natural gas (LNG) production and air separation unit (ASU) is recommended to cover any loss during the purification process [117].

### 3.2. Anti-Sublimation (AnSU)

**Figure 5:**
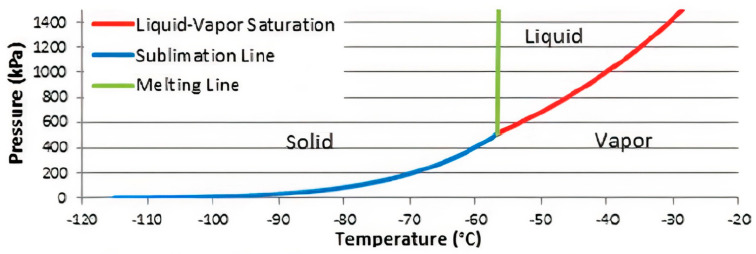
CO_2_ phase diagram [119]. Reprinted with permission from Pan, X., Clodic, D. and Toubassy, J. (2013). Copyright 2021 John Wiley and Sons.

Anti-sublimation (AnSU) is a phrase referring to the reverse phase change from gas to solid, where the initial term sublimation is defined as the phase change from solid to gas. CO_2_ removal using AnSU is a post-treatment method that utilizes the thermodynamics of CO_2_ anti-sublimation at atmospheric pressure [118]. CO_2_ is directly converted from gas to solid phase at a point when the pressure is lower than the triple point pressure. Figure 5 shows the phase diagram for pure CO_2_ that illustrates the CO_2_ phase change with respect to pressure and temperature.

**Figure 6:**
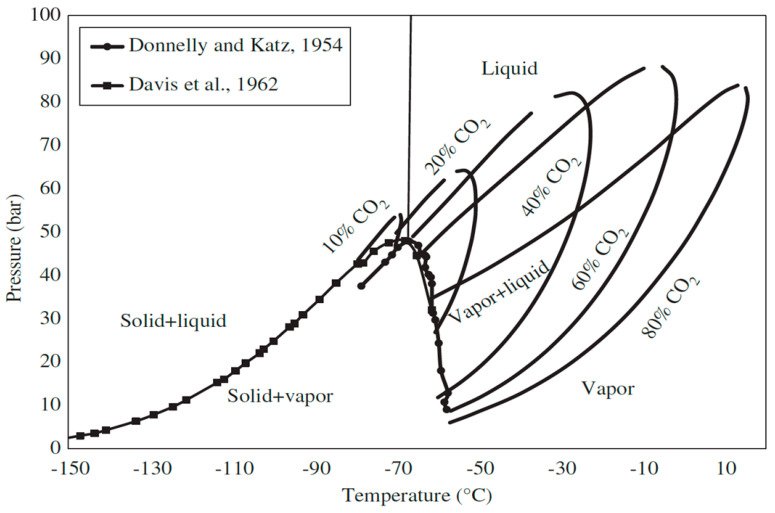
Phase envelope of CO_2-_CH_4_ mixture [120]. Reprinted with permission from Maqsood, K. et al. (2015). Copyright 2021 Walter de Gruyter and Company.

The sublimation temperature of CO_2_ also depends on its concentration inside the flue gas. The flue gas needs to further cool down to bring the CO_2_ into the solid phase if the CO_2_ pressure is lower than its triple point. Figure 6 shows the phase envelope for the CO_2_ and CH_4_ mixture with variation of the CO_2_ concentration. The triple point for every CO_2_ and CH_4_ mixture varies depending on the CO_2_ concentration. For instance, the triple point of 80% of CO_2_ has been found at −65 °C and 30 bar of pressure. Based on Figure 6, the anti-sublimation process needs to maintain pressure between 1 bar and 40 bar and temperature between −60 °C and −150 °C for obtaining the solid–vapour phase, depending on the CO_2_ concentration [120]. Consequently, the CH_4_ which is still in vapour form can be separated and commercialised.

**Figure 7:**
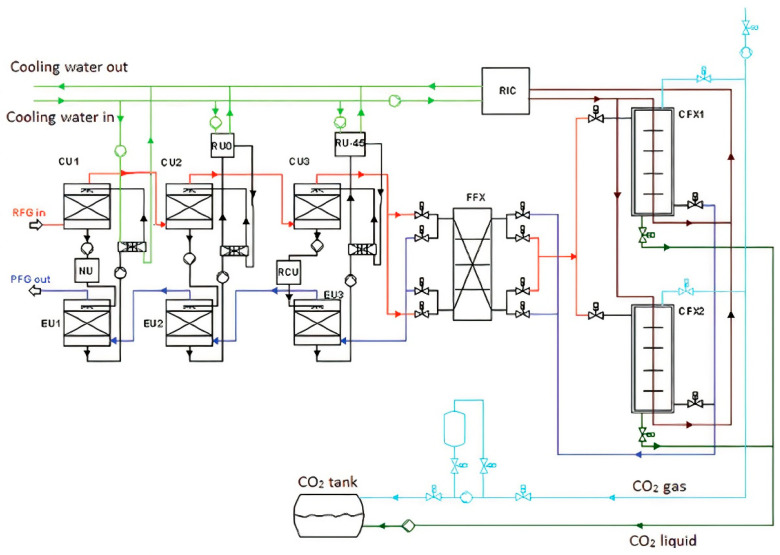
Schematic diagram of anti-sublimation process mechanism [119]. Reprinted with permission from Pan, X., Clodic, D. and Toubassy, J. (2013). Copyright 2021 John Wiley and Sons.

There are five stages in the AnSU process for CO_2_ removal as illustrated in Figure 7. The first stage is mainly for moisture removal and cooling by lowering the temperature down to −40 °C. The removal of water in this stage is crucial to avoid any hydrates in the main process line later. There are three loops of water removal set in series which consist of a condensing unit (CU) and evaporating unit (EU) for each loop. The rich flue gas (RFG) is fed into CU1, CU2 and CU3 and cooled by the sprayed solution during these three stages of condensing units. Meanwhile, the poor flue gas (PFG) goes to three evaporation units (EU), which are EU1, EU2 and EU 3. The solution loops are heated by RFG in CU and cooled by PFG in EU. This solution mainly consists of CaCl_2_ to capture the water. The temperature of the flue gas after CU1 is about 20 °C and 0 °C after CU2 with a water content of 6500 ppm. After CU3, the sprayed CaCl_2_ reduces the temperature down to −40 °C with a water content of 160 ppm.

The second stage of AnSU is mainly to reduce the water content down to 1 ppm level. The heat exchanger (FFX) is installed after CU3 and PFG goes to EU3 to recover the coldness of PFG coming from the low-temperature CFX. RFG is cooled down to −100 °C in FFX. The defrosting approach at FFX is important, as the water content at the RFD inlet is 160 ppm and targeted to obtain 1 ppm at the RFG outlet. The third stage of AnSU is to provide a low temperature for CFX to convert CO_2_ from gas to solid. The RIC consists of several cooling stages.

The fourth stage is the CO_2_ freezing in the CFX heat exchanger. RFG enters CFX at −100 °C and the CO_2_ will antisublimate, where the CO_2_ phase directly converts into solid from gas. CFX is designed to present a temperature glide along the section since the CO_2_ partial pressure of 12 kPA will solidify at −101 °C and −119 °C at 1.5 kPa. The final stage of the AnSU process is CO_2_ recovery. The CO_2_ is not only covered in liquid but also gas from the RIC evaporator enclosure. The major advantage of this AnSU technology is that high purity of CO_2_ captured (99.9% purity) is obtained without having any contamination [119,121,122,123].

A few studies show that AnSU technologies could provide less energy consumption compared to the typical absorption technology in terms of CO_2_ removal. A study performed by D. Clodic et al. stated that the energy consumption for CO_2_ removal is only high at a low CO_2_ concentration and becomes lower as the concentration of CO_2_ is increased [123]. Hence, the AnSU can be implemented either in low or high CO_2_ concentrations, as the energy consumption for CO_2_ concentration above 10% is significantly low.

Schach et al. studied and compared AnSU and adsorption technology in terms of electrical energy consumption using ASPEN HYSYS for the same 90% of CO_2_ removal efficiency [124]. They found that AnSU technology is better at reducing electrical energy demand compared to the adsorption of CO_2_ using MEA. The total electrical power required for AnSU, which is 178 MW, is much lower, while the adsorption is 209 MW. Moreover, the specific electrical energy required per kg of CO_2_ captured in AnSU technology is also lower compared to the adsorption, with values of 0.286 kWh/kg CO_2_ and 0.391 kWh/kg CO_2,_ respectively, which reduces the total operation expenditure (OPEX) for the treatment plant [124]. Table 2 illustrates the summary of the comparison between AnSU and adsorption technologies performed by Schach et al.

Even though AnSU is a great technology for high efficiency and as an alternative for CO_2_ removal, this technology faces several limitations and challenges in terms of operation and maintenance. The major drawback for AnSU is the usage of several compression systems in the process which require regular maintenance due to involvement of rotating parts. This compression system is essential to provide the expansion condition that will be used for obtaining the lower temperature due to Joule–Thomson effect. The regular maintenance for this compression system will increase the operation expenditure from time to time. In addition, the capital expenditure for AnSU is also relatively higher due to its complexity to operate, which was discussed previously in the mechanism of AnSU. AnSU consists of five stages of process mechanisms in which each stage has its own special required equipment. However, there is no study yet on simultaneous CO_2_ and H_2_S removal that utilises AnSU as one of the removal techniques. To overcome the limitations and challenges faced by AnSU, it is proposed to integrate cooling energy from other sources to the heat exchanger inside the AnSU process. A good supply of cooling energy could replace the complexity of the compression and expansion systems in providing a low temperature for the system.

### 3.3. Controlled Freeze Zone (CFZ)

The application of the conventional cryogenic distillation process in gas separation, particularly involving CO_2_, was often subjected to CO_2_ solidification at low temperatures [125]. Intensive efforts have been devoted to valorize the solidified CO_2_ obtained from the cryogenic separation for geo-sequestration of CO_2_. In 1985, ExxonMobil Upstream Research Company introduced and patented a nonconventional cryogenic technology of controlled freeze zone (CFZ^TM^) technology to separate CO_2_ and H_2_S from natural gas [125,126]. CFZ^TM^ technology is usually used for bulk CO_2_ removal from natural gas. This technology offers an integrated solution to overcome the bottlenecks of cryogenic technology through a single-step cryogenic distillation process to treat sour gas resources with high contaminants such as CO_2_. Compared to the conventional cryogenic approach, the CFZ^TM^ process allows CO_2_ to freeze out under controlled conditions using a unique designed section in the distillation column. A typical CFZ^TM^ process with a modified rectification section is capable of purifying natural gas to meet pipeline specifications with CO_2_ concentration of less than 50 ppm [127]. CFZ™ offers lower capital expenditure (CAPEX) compared to the other conventional separation technologies, owing to its small footprint, less processing equipment and no solvent being required.

**Figure 8:**
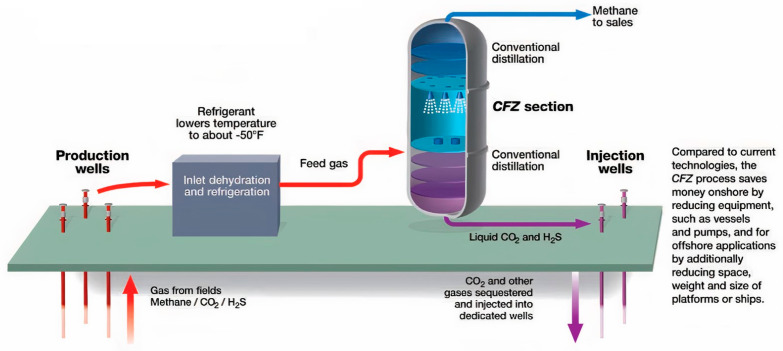
Process flow for controlled freeze zone (CFZ^TM^) cryogenic distillation technology [125]. Reprinted with permission from Abdulsalam, J. et al. (2018). Copyright 2021 Taylor & Francis.

CFZ™ usually operates at a constant pressure while the operating temperature is determined by the feed conditions and expected product specifications. The configuration of CFZ^TM^ technology is illustrated in Figure 8. The CFZ^TM^ tower is comprised of three sections: (1) the conventional upper rectifying section, (2) the controlled freeze zone section and (3) the conventional for bottom stripping section [128]. At the upper section, CO_2_ content is reduced in the rectifying section via conventional distillation. Entering the solidification area, the liquid from the upper section is sprayed into the unobstructed opening area of the CFZ™ section through nozzles where it encounters a warmer temperature ranging between −90 °C and −85 °C and vaporizes lighter components such as CH_4_. Consequently, the liquid droplet is increasingly rich with CO_2_ and solidified within the chamber at a solidification temperature of −62 °C to −45 °C. Beyond the solidification condition, the solids CO_2_ forms fall onto the liquid layer at the bottom of the CFZ™ section and are directed to the stripping section for CH_4_ recovery. CH_4_ is stripped off from the bottom liquid stream enriched with CO_2_ and other contaminants at the bottom of the CFZ™ chamber via conventional distillation. Besides CO_2_, the bottom liquid stream may also contain other contaminants such as H_2_S, mercaptans and heavy hydrocarbons. As a result, the top product exiting the column is significantly concentrated with CH_4_.

In 1985, Exxon Production Research built a pilot plant with a capacity of 0.6 MCFD at Clear Lake Gas Plant, Houston to evaluate the capability of this technology to process natural gas containing CO_2_ between 15% and 65%. The CFZ™ was operated at pressure ranging from 3447 to 4137 kPa. The pilot plant successfully obtained products that met pipeline quality which approached LNG specification by reducing CO_2_ content down to ppm levels along with a minimum CH_4_ loss of 0.5%. Following the CDP, ExxonMobil developed a commercial demonstration project (CDP) in 2007 at Shute Creek Gas Treating facility (SCTF), LaBarge, WY, USA to evaluate the commercial readiness of CFZ™ technology. The project aimed to process and sequester CO_2_ from sour natural gas for acid gas injection application [129]. The CDP was designed to process 228.89 m^3^/s natural gas with CO_2_ and H_2_S content of 65% and 5%, respectively, at the high pressure of 4137 kPa [130]. This process is capable of obtaining the overhead product with CO_2_ and H_2_S composition of 680 ppm and 1.2 ppm, respectively. Moreover, the bottom liquid product contains 4.1% CO_2_ and 0.23% H_2_S. The energy requirement of CFZ™ technology is not yet reported elsewhere.

Interestingly, the application of CFZ™ technology is economically viable for highly sour gas separation because it is capable of processing a wide range of CO_2_ and H_2_S compositions in the feed gas. The overhead product and bottom liquid product, which are discharged at relatively high pressure, significantly reduce the recompression cost for sales gas delivery through pipeline and acid gas reinjection to the reservoir. To date, acid gas injection (AGI) emerged as a common approach to dispose the separated CO_2_ and H_2_S from natural gas. The combination of CFZ™ and AGI technology facilitates the geo-sequestration of CO_2_ for enhanced oil recovery by exploiting the high-pressure liquid stream discharged at the bottom of the column. On the other hand, the CFZ™ process also imposed a less stringent dehydration requirement rendered by the high water-holding capacity of the liquid CO_2_ [131]. Valencia et al. [131,132] reported that compared to Ryan Holmes and combined bulk fractionation-Selexol processes, CFZ™ demonstrated lower capital expenditure by 5% and 10%, respectively. Mart [133] cited that the economic advantages offered by CFZ™ are attributed to the simplicity of the process that minimizes the needs of processing equipment. Using CFZ™, a significant operational cost–savings between 12% and 37% was attained, relative to the conventional processes such as Selexol, combined bulk fractionation-Selexol- and Ryan Holmes. Furthermore, a greater sales gas revenue of 4% to 8% can be achieved by exploiting the excellent process efficiency through the integrated configuration of CFZ™ and AGI technologies. However, the application of CFZ™ for simultaneous separation of CO_2_ and H_2_S remains challenging due to the inhibition of CO_2_ solidification with the presence of H_2_S [130]. Currently, the demonstration project of CFZ™ has been completed and the technology is ready for scale-up and commercialization to process sour feed gas up to 326.99 m^3^/s [134].

### 3.4. CryoCell

CryoCell^®^ is a nonconventional cryogenic technology developed by Cool Energy Ltd. in 2009 to treat high-CO_2_ natural gas fields using the similar concept of CO_2_ sublimation as applied in CFZ™ process [135]. The portable and compact design of CryoCell^®^ provides the flexibility of this process for offshore applications [136]. Besides separation, this technology also demonstrates great potential for geo-sequestration of CO_2_ by reinjecting the liquid CO_2_ obtained in the bottom product into the reservoir for geological storage. Compared to the conventional separation technology, CryoCell^®^ offers economic advantages by eliminating the need for water consumption, chemicals or solvents and minimizing the possibilities of corrosion-related issues. These benefits would result in lowering capital cost of CryoCell^®^ by 20% to 40% relative to the conventional LNG purification process. In fact, by taking advantage of the Joule–Thomson effect, the requirement for the refrigeration process can be significantly minimized. The laboratory trials of CryoCell^®^ demonstrated the capability of this process to reduce up to 70% of CO_2_ content in natural gas and moisture levels down to less than 200 ppm.

**Figure 9:**
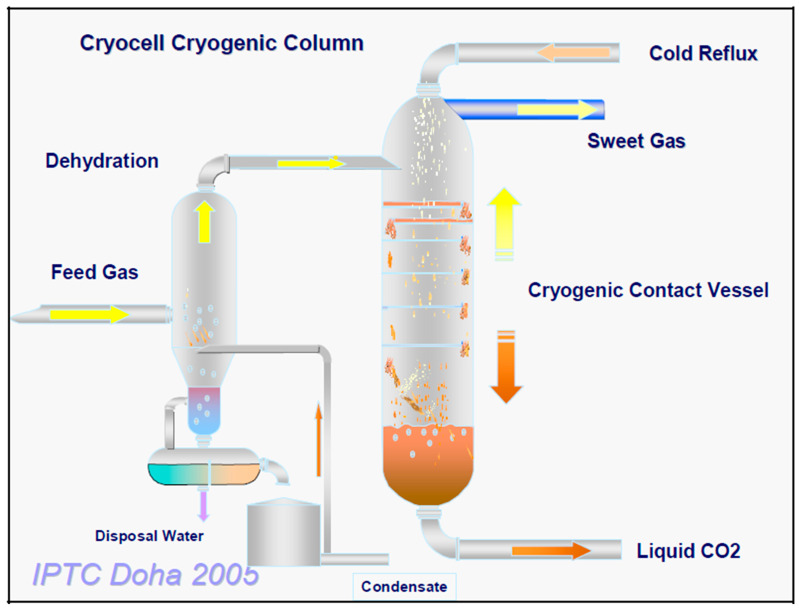
Process flow for CryoCell^®^ technology [136]. Reprinted with permission from Amin, R., Jackson, A. and Kennaird, T. (2005). Copyright 2021 Society of Petroleum Engineers.

The configuration of the CryoCell^®^ process is illustrated in Figure 9. In the CryoCell^®^ process, the feed gas containing high CO_2_ initially undergoes a dehydration process to reduce the moisture level down to 5 ppm [110]. The dried feed gas is then cooled to a temperature higher than the freezing point of CO_2_ at a constant pressure, which results in condensation of the feed gas mixture into liquid phase. Next, the liquid mixture expands through the Joule–Thomson valve at a constant enthalpy that leads to phase change of the CO_2_ into three phases that include liquid, solid and vapor [137]. The operating condition of CryoCell^®^ is critical to ensure low CO_2_ content in the vapor phase while maintaining rich CO_2_ in the liquid product. The different phases of CO_2_ are separated in the CryoCell^®^ separator. The solid CO_2_ collected at the bottom of the separator is melted using external heating sources and combined with the existing liquid product. Besides CO_2_, the solids product may also contain heavy hydrocarbons and H_2_S. The rich-CO_2_ liquid product that exits the bottom of the separator is compressed to meet the required disposal pressure prior to the reinjection into the reservoir. Meanwhile, the vapor product emerging from the top of the column is compressed to meet sales gas delivery specifications.

In 2006, a commercial demonstration plant (CDP) was developed by Cool Energy Ltd. in collaboration with Shell Global Solutions at Perth Basin, Western Australia to explore the viability of this technology. The plant design of this 2 MMscf/d plant was built based on the process flow scheme for low CO_2_-lean gas as proposed by Hart and Gnanendran [135]. The compact module of CryoCell^®^ was designed to process natural gas with CO_2_ content up to 60 mol% at feed pressure ranging between 5500 and 6500 kPag. The feed flow rate also varies between 600 kg/h and 1300 kg/h based on the desired feed stream composition. The CryoCell^®^ separator was operated at three different pressures of 1200 kPag, 1600 kPag and 1900 kPag. Meanwhile, the temperature of the reboiler was maintained between −50 °C and −60 °C throughout the testing. The CDP revealed that the CryoCell^®^ technology successfully reduced about 81% CO_2_ in natural gas.

Hart and Gnanendran [135] performed a benchmark study between CryoCell^®^ versus amine process which aimed to treat 16.5 m^3^/s feed gas with CO_2_ composition of 20 mol% and 35 mol% using Aspen HYSYS. Based on the simulation, CryoCell^®^ utilized significantly lower heat duty of less than 0.1 MW compared to the amine process which required process heating between 19 and 35 MW. In view of economics, CryoCell^®^ technology demonstrated significant cost savings for plant installation with the total cost of 65.84 AUD/kg of CO_2_ and 90.88 AUD/kg of CO_2_ for 20 mol% and 35 mol% of CO_2_ content, respectively. Meanwhile, the amine process yielded a total cost of 86.70 AUD/kg of CO_2_ and 146.53 AUD/kg of CO_2_ for both CO_2_ contents. This economic benefit exploits the advantages of the simplified gas treatment process, geo-sequestration of CO_2_ and low energy requirement due to elimination of solvent pumping and heat requirement for the reboiler duty. Nonetheless, the compression and refrigeration requirement slightly offset the cost–savings of CryoCell^®^ technology. The simulation data show that CryoCell^®^ technology requires higher compression power, between 4.3 MW and 7.0 MW, than the amine absorption process, which requires between 1.9 MW to 3.8 MW, at increasing CO_2_ content in natural gas.

Cool Energy Ltd. is expected to undertake the commercialization of this technology in the future with the successful completion of the first phase of Front-End Engineering and Design (FEED) study for 19.62 m^3^/s Cryocell^®^ plant at Cooper Basin, South Australia. Provided with the commercial demonstration plant testing and simulation data, CryoCell^®^ shows a huge potential for natural gas treatment with high CO_2_ while sequestering the CO_2_ for geological storage. Currently, the studies on the application of CryoCell^®^ are still ongoing to overcome the operational challenges to control the CO_2_ freezing and handling of solids formation. According to Amin et al. [136], without reliance on any pretreatment system such as amine or membrane process, CryoCell^®^ is capable of handling a wide range of contaminants such as heavy hydrocarbons and H_2_S. However, limited literature is available to further understand the application of this technology for H_2_S removal. Hence, future work that extends on this application would be beneficial to monetize the underdeveloped sour gas fields by taking advantage of their benefits in terms of energy requirement, economics and compact design.

## 4. Future Outlooks and Perspectives of Cryogenic Technology

The exploration of sour natural gas fields plays a significant role in ensuring a sustainable energy supply to accommodate the rising energy demand fueled by the growth of population and economy. From the viewpoint of economics, cryogenic technology is well-suited for bulk CO_2_ removal to monetize the subquality gas fields. In fact, the technology also demonstrates a great potential towards achieving the Net Zero Carbon Emission 2050 aspiration. Despite the excellent advancement of the cryogenic process, the current application of cryogenic separation is usually limited to CO_2_ separation, while removal of H_2_S is still rarely reported. The removal of H_2_S through the cryogenic process remains challenging, as it is a highly energy-intensive process which requires a very low operating temperature, leading to dramatic increases in operating expenditure [110]. Hence, future work on the integration between cryogenic process and liquefied natural gas (LNG) production is significantly important to harness cold energy to provide a sufficiently low temperature for the cryogenic separation. Consequently, the operating expenditure of cold utilities and energy consumption can be significantly minimized. Moreover, in terms of thermodynamics, the phase equilibrium data are typically available for CO_2_–CH_4_ binary gas systems, while the presence of higher hydrocarbons and contaminants are rarely highlighted. Therefore, further research is necessary to develop a thermodynamic model which accounts for the multicomponents present in natural gas [138,139]. The thermodynamic study is crucial to facilitate the design and optimization of an energy-efficient and high-performance cryogenic process. On the other hand, hybrid methods which integrate the cryogenic process and other separation processes, such as membrane separation and absorption, are identified as promising approaches to enhancing the efficiency of natural gas purification. By exploiting the advantage of high CO_2_ recovery, hybrid processes are anticipated to produce high-quality LNG that meets market specification with lower energy consumption [140].

## 5. Conclusions

The criticality of CO_2_ and H_2_S removal from natural gas which aims to monetize undeveloped sour gas fields is highlighted in this review paper. The application of the conventional separation technologies for natural gas sweetening such as absorption, adsorption and membrane and cryogenic separation are briefly discussed. However, these conventional technologies are often associated with multiple drawbacks such as energy-intensiveness, corrosion issues, high compression and regeneration cost, as well as degradation of materials’ integrity due to long operation time. In fact, these technologies were also reported less economical for the treatment of highly sour natural gas. Thus, cryogenic separation emerged as an economically attractive technology for natural gas upgrading, particularly for feed gas with high CO_2_ content. Multiple benefits are offered by this technology in terms of capital and operational cost, energy requirements, product purity and environment. Until now, several unconventional cryogenic technologies, such as cryogenic packed bed, anti-sublimation (AnSU), Controlled Freeze Zone (CFZ™) and CryoCell^®^, are currently explored to enhance the efficiency of the cryogenic separation. In addition, these nonconventional cryogenic technologies also offer huge potential in the sequestration of CO_2_ for enhanced oil recovery and minimization of greenhouse gas effects. The commercial readiness of cryogenic technology is also reviewed in the present work. While substantial research and commercial trials of the different cryogenic technologies are currently available for high CO_2_ removal, the data for H_2_S removal are still scarce. Thus, future work is necessary to fully comprehend the viability of these technologies for upscaling and commercialization for coremoval of CO_2_ and H_2_S application.

## Figures and Tables

**Table 1:** Important insights of the review.

Category	Insights
Conventional CO_2_ and H_2_S separation technologies	Gas separation mechanisms of the conventional technologies—absorption, adsorption and membranes and cryogenic separation. Research and development of technologies for sour natural gas treatment Challenges or limitations of conventional separation technologies
Advanced cryogenic distillation process	Modifications or advancements in the conventional cryogenic process—cryogenic packed bed, anti-sublimation, controlled freeze zone (CFZ) and CryoCell The extent of the capability of the processes to handle bulk CO_2_ and H_2_S in sour natural gas State-of-the-art and perspective of the advanced cryogenic process for natural gas upgrading application

**Table 2:** Power plant efficiency loss, total power required and specific electric required comparison between adsorption and AnSU [124]. Adapted with permission from Schach, M. et al. (2011). Copyright 2021 Elsevier.

Technology Used	Adsorption	AnSU
Power plant efficiency loss	12.5%	10.7%
Total electrical power required	209 MW	178 MW
Specific electric required	0.391 kWh/kg CO_2_	0.286 kWh/kg CO_2_

## Data Availability

Not applicable.

## Sample Availability

Samples of the compounds are not available from the authors.
